# Circular RNA circ_0001006 aggravates cardiac hypertrophy via miR-214-3p/PAK6 axis

**DOI:** 10.18632/aging.203461

**Published:** 2022-03-06

**Authors:** Xuefeng Lin, Liqin Zhang, Wei Zhang, Xinjun Lei, Qun Lu, Aiqun Ma

**Affiliations:** 1 Department of Cardiovascular Medicine, The First Affiliated Hospital of Xi’an Jiaotong University, Xi’an, Shanxi 710061, PR China; 2 Key Laboratory of Molecular Cardiology, Xi’an Jiaotong University, Xi’an, Shanxi 710061, PR China; 3 Institute of Cardiovascular Channelopathy, Xi’an Jiaotong University, Xi’an, Shanxi 7100161, PR China; 4 Key Laboratory of Environment and Genes Related to Diseases, Xi’an Jiaotong University, Ministry of Education, Xi’an, Shanxi 7100161, PR China; 5 Department of Cardiovascular Medicine, The First Affiliated Hospital of Baotou Medical College, Baotou, Inner Mongolia 014010, PR China; 6 Baotou Medical College, Baotou, Inner Mongolia 014010, PR China; 7 ECG Room, Xi’an Children’s Hospital, Xi’an, Shanxi 710061, PR China

**Keywords:** circular RNAs, cardiac hypertrophy, miRNA, gene expression, heart disease

## Abstract

Aim: Circular RNAs (circRNAs) control gene expression in a series of physiological and pathological processes, but their role in heart disease is unknown. This research illustrates the role and potential mechanism of circRNA in cardiac hypertrophy.

Methods and Results: In this report, we found that circular RNA hsa_circ_0001006 (circ_0001006) was upregulated in cardiac hypertrophy mice and cardiomyocytes treated with angiotensin II (Ang II). Next, we noticed that gain of function circ_0001006 could induce cardiomyocyte hypertrophy; oppositely, knockdown of circ_0001006 remitted Ang II-induced cardiomyocyte hypertrophy. Biotin-coupled miRNA and RNA-pull down assays showed that miR-214-3p could bind with circ_0001006 and gain the function of miR-214-3p abrogated the pro-hypertrophy effect of circ_0001006. Furthermore, Further, dual-luciferase reporter assay showed that miR-214-3p could interact with 3′UTRs of the PAK6 gene, and circRNA_0001006 could block the above interactions. Additionally, PAK6 expression is inhibited by miR-214-3p mimic in cardiomyocytes but enhanced by over-expression of circRNA_000203 *in vitro*.

Conclusions: Our data demonstrated that circRNA_0001006 exacerbates cardiac hypertrophy via suppressing miR-214-3p leading to enhanced PAK6 levels.

## INTRODUCTION

Cardiac hypertrophy can be divided into physiological and pathological types related to normal cardiac function and impaired cardiac function [1, 2]. Pathological hypertrophy of the myocardium is associated with several adverse cardiovascular events, including arrhythmias and heart failure. Studies have shown that chromatin remodeling and histone modification play essential roles in myocardial development and heart disease [3]. Non-coding RNAs are critical participants in cardiovascular physiological and pathological processes [4]. One of the non-coding RNAs, miRNAs, are 20–23 nucleotide RNAs that mediate various physiological functions and diseases by regulating target genes. Previous reports showed that miR-498 [5], miR-206 [6], miR-155 [7], and miR-451 [8] were all involved in cardiac hypertrophy progress.

Circular RNA (circRNA) serves as an essential class of non-coding RNA (ncRNA) and is mainly produced by reverse splicing of mRNA from the 3′ and 5′ end precursors of thousands of exons in eukaryotes [9, 10]. Increasing evidence has identified that circRNAs possess the binding sites of miRNAs and function as sponges of miRNAs to regulate target genes [11, 12]. CircRNA is involved in a variety of heart disease processes [10, 13]. Pan RY et al. found that 66 circRNAs are involved in the regulation of coronary artery atherosclerosis and may be applied as the therapeutic or diagnostic markers [14, 15]. ATP2A2 performed a ceRNA relationship with hsa_circ_0046159, which could regulate chronic thromboembolic pulmonary hypertension within the miR-1226-3p-circRNA regulatory network. [16]. Circ-HIPK3 promoted function of adrenaline via controlling miR-17-3p binding with ADCY6 during heart failure [17]. In addition, CircFndc3b modulates FUS/VEGF-A axis after myocardial infarction and performs a protection effect [18].

Here, we performed that circ_0001006 was induced in cardiac hypertrophy mice and Ang II-treated cardiomyocytes, which displayed pro-hypertrophy effect by inhibiting miR-214-3p and demonstrated a mechanism of circ_0001006 via the miR-214-3p/PAK6 axis.

## RESULTS

### Upregulation of circ_0001006 in cardiac hypertrophy mice and AngII-treated cardiomyocytes

**Figure 1:**
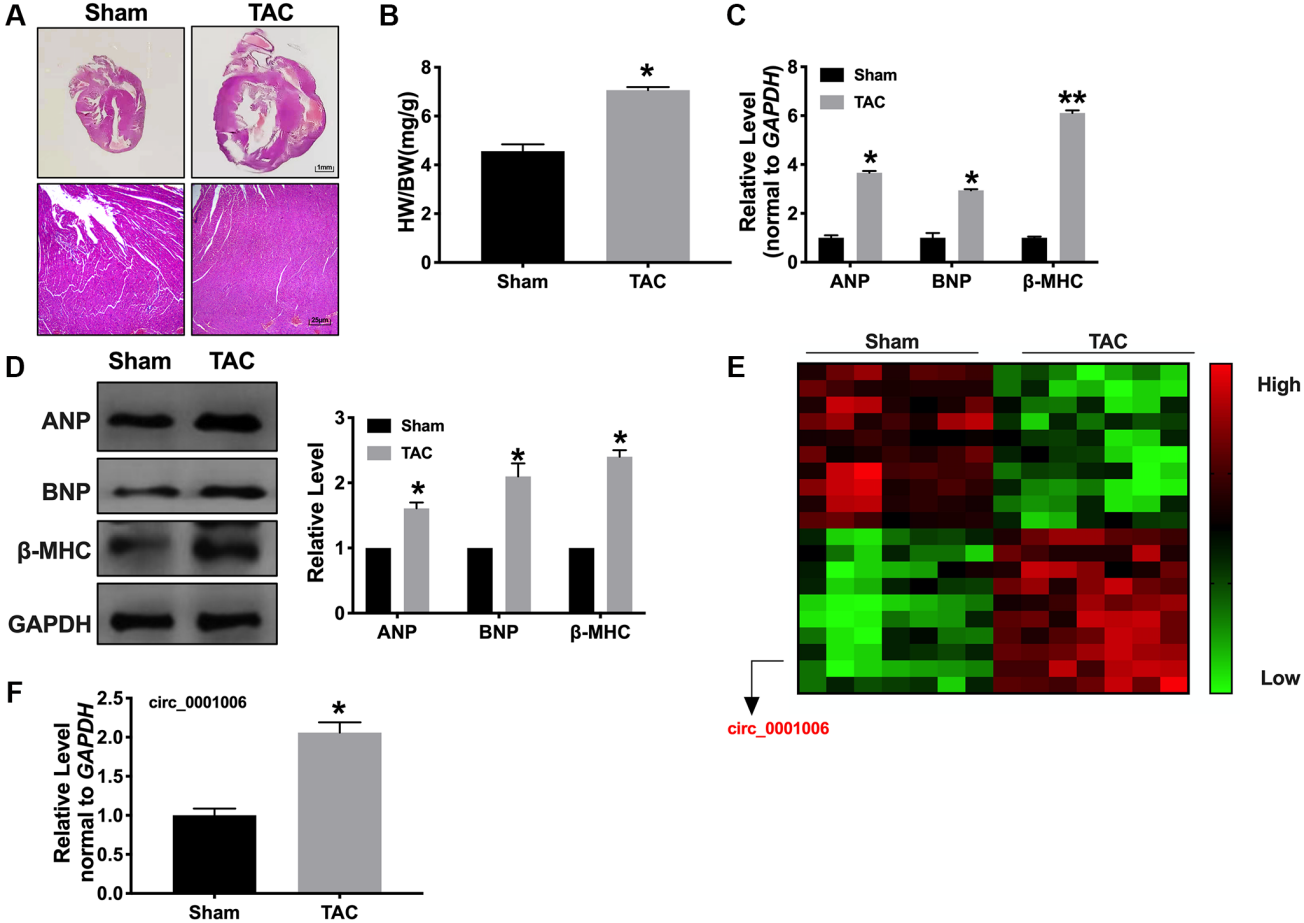
**Upregulation of circ_0001006 in TAC induced cardiac hypertrophy.** (**A**) Morphological experiment was performed to visualization the heart size (H&E staining, Sham indicated sham-operated group, TAC indicated transverse aortic constriction operation group). (**B**) The histogram results of HW/BW ratios in a different group. (**C**) qRT-PCR assays of cardiac hypertrophy biomarker mRNA level, ANP, BNP, and β-MHC. (**D**) The expression of circ_0001006 in TAC and Sham mice. (**E**) The microarray of circRNAs in TAC and Sham mice tissues. (**F**) qRT-PCR assay analysis of the circ_0001006 expression level in heart tissues. ^*^P < 0.05 and ^**^P < 0.01. All experiments were repeated at least three independent experiments.

Firstly, the mice were randomly divided into two groups, including sham and TAC group. The TAC group performed the ventricles wall thickened. We also overserved the enhanced heart weight to body weight (HW/BW) ratio (Figure 1A and 1B). We observed the expression of the biomarkers indicating hypertrophy, such as ANP, BNP, and β-MHC, by RT-PCR and Western blot assays. The result showed an increased level in the TAC group compared with the sham group (Figure 1C and 1D). Then, a microarray was performed to screen the differential expression of circRNA. Among them, circ_0001006 was the most significant change in TAC tissues (Figure 1E). RT-PCR assays results were also performed that circ_0001006 was upregulated in the TAC group (Figure 1F).

**Figure 2:**
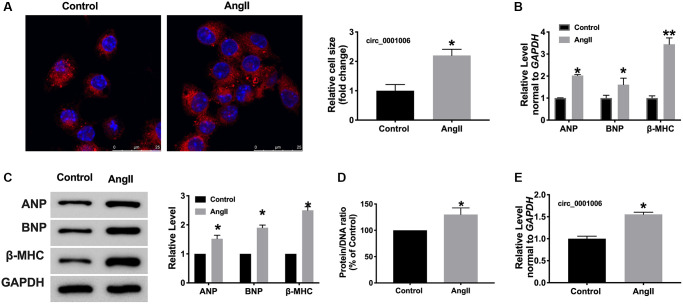
**circ_0001006 was upregulated in AngII-induced cardiomyocyte hypertrophy.** (**A**) The immunofluorescence results of cardiomyocytes treated with or without Ang II. (α-actinin, red, DAPI, blue, The scale bar is 25 μm). (**B**) qRT-PCR assay analysis of the circ_0001006 expression level in cardiomyocytes. (**C**) Western blot analysis of ANP, BNP, β-MHC expression in cardiomyocytes. (**D**) Protein/DNA ratio of the cardiomyocytes. (**E**) The expression of circ_0001006 *in vitro*. ^*^P < 0.05 and ^**^P < 0.01. Data are shown as mean ± SEM. All experiments were repeated at least three independent experiments.

Furthermore, A *vitro* model was used in this research. Cardiomyocytes were treated with Ang II (200 nmol/L) for 48 hrs, both cell cross size, hypertrophic biomarkers, and protein/DNA ratio increased remarkably in Ang II-treated cells (Figure 2A–2D). Consistent with results *in vivo*, circ_0001006 was upregulated in cardiomyocytes treated with Ang II (Figure 2E).

### Circ_0001006 induces hypertrophy of cardiomyocytes

**Figure 3:**
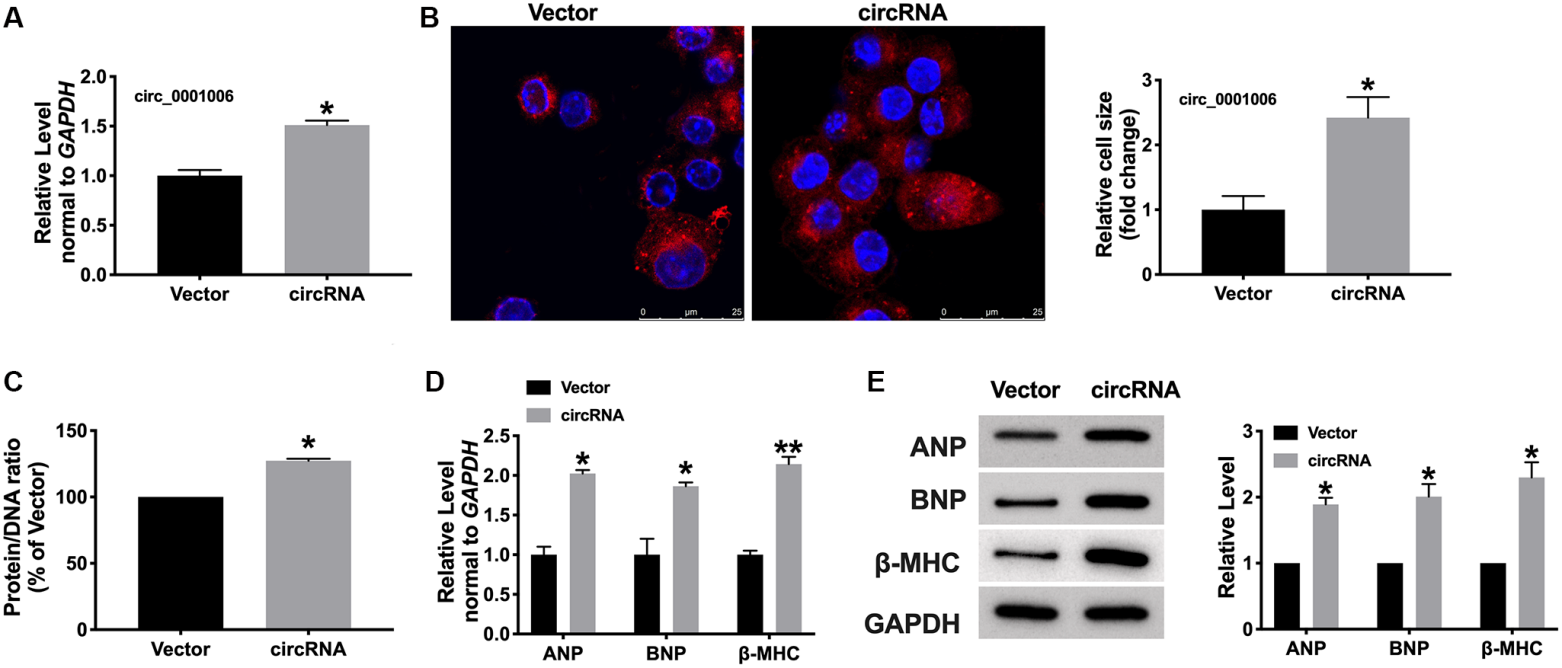
**circ_0001006 induced hypertrophic in cardiomyocytes.** (**A**) The level of circ_0001006 in cardiomyocytes after transfecting circ_0001006/Vector. (**B**) Representative immunofluorescence results of cardiomyocytes after transfecting circ_0001006/Vector (The scale bar is 25 μm). (**C**) Protein/DNA ratio of the cardiomyocytes after transfecting circ_0001006/Vector. (**D**) The mRNA level of ANP, BNP, and β-MHC in cardiomyocytes. (**E**) Western blot assay of ANP, BNP, β-MHC protein level in cardiomyocytes. ^*^P < 0.05 and ^**^P < 0.01. Data are shown as mean ± SEM. All experiments were repeated at least three independent experiments.

We transfected the constructed plasmid circ_0001006 and vector into cardiomyocytes, the efficiency of overexpression was about 51.22 % (Figure 3A). Fluorescence assays displayed the enlarged cell size induced after transfection circ_0001006 (Figure 3B), overexpression of circ_0001006 resulted in increases in the protein/DNA ratio (Figure 3C). Furthermore, the hypertrophic markers were all increasing compared with the vector group, which indicated the pro-hypertrophy effect of cardiomyocytes (Figure 3D and 3E). In summary, overexpression of circ_0001006 would induce cardiac hypertrophy.

### Knockdown of circ_0001006 remits myocardial hypertrophy

**Figure 4:**
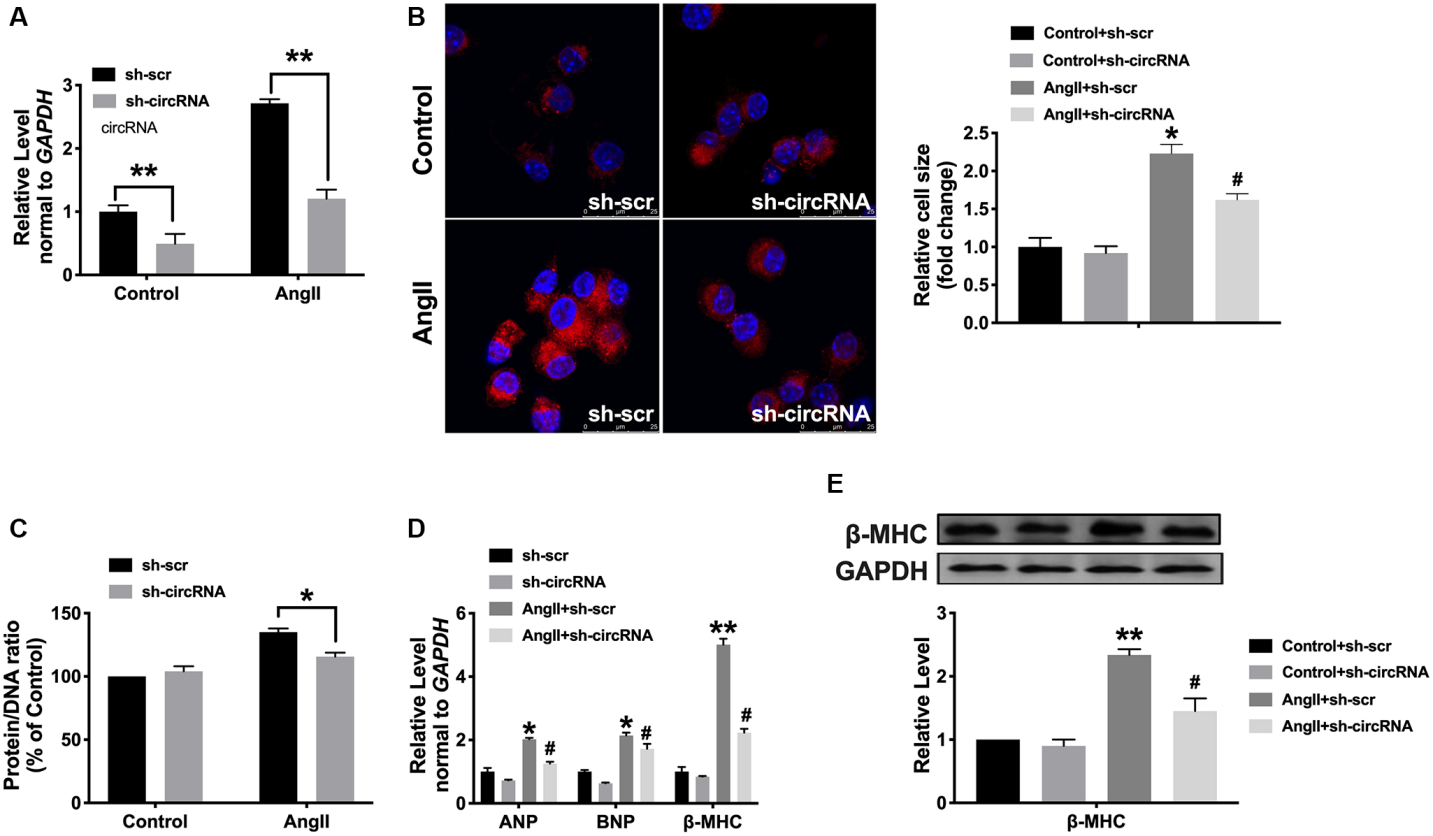
**Silencing of circ_0001006 prevents cardiomyocyte hypertrophy.** (**A**) The transfection efficiency of sh-circ_0001006 was verified. (**B**) Representative immunofluorescence results performed the effect of knocking down circ_0001006 (The scale bar is 25 μm). (**C**) Protein/DNA ratio of the cardiomyocytes after transfecting with sh-circ_0001006/sh-Scr treated with or without Ang II. (**D**) ANP, BNP, and β-MHC expression level in cardiomyocytes. (**E**) The expression of β-MHC in cardiomyocytes. ^*^P < 0.05 and ^**^P < 0.01 vs. Control+sh-scr group, ^#^*P* < 0.05 vs. Control+sh-circRNA. Data are shown as mean ± SEM. All experiments were repeated at least three independent experiments.

Then, we would like to determine whether the loss function of circ_0001006 performs inhibition function on cardiac hypertrophy, and we, therefore, created short hairpin RNA (shRNA) sh-circ_0001006 or sh-Scramble (sh-Scr) and transfected into cardiomyocytes treated with Ang II (200 nmol/L) for 48 hrs (Figure 4A). The cell surface area was performed by immunofluorescence (Figure 4B), loss function of circ_0001006 decreased cell cross area and the protein/DNA ratio after disposing of Ang II (Figure 4C), the levels of hypertrophic markers were also reduced compared with the sh-Scr group (Figure 4D and 4E).

### MiR-214-3p/PAK6 may be downstream of circ_0001006

**Figure 5:**
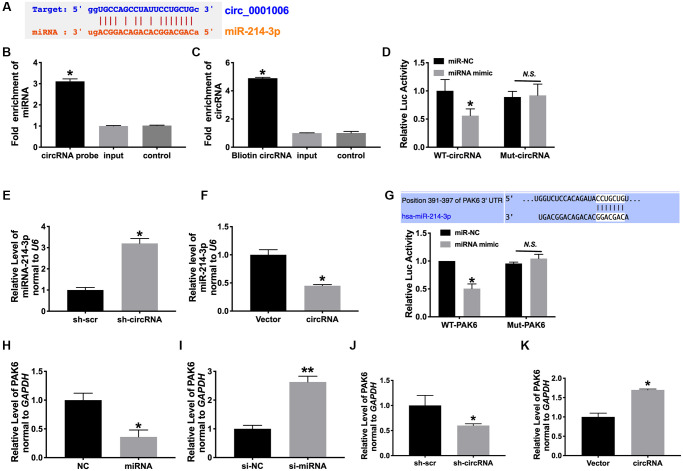
**MiR-214-3p/PAK6 may be downstream of circ_0001006.** (**A**) Bioinformatics website forecast revealed that circ_0001006 includes site binding to miR-214-3p provided by predication tools (**http://starbase.sysu.edu.cn/index.php**). (**B**) circ_0001006 in cell lysis was pulled down and collected with a circ_0001006-specific probe and then measured using qRT-PCR. miR-214-3p was pulled down and collected with a circ_0001006-specific probe and then evaluated using qRT-PCR. (**C**) Luciferase assay report verified the relationship between circ_0001006 and miR-214-3p. (**D** and **E**). the expression of miR-214-3p in cardiomyocytes after sh-circRNA and circRNA transfection. (**F**) Luciferase assay report verified the relationship between PAK6 and miR-214-3p. (**G**–**K**). The expression of PAK6 was detected in cardiomyocytes. Data are shown as mean ± SEM. ^*^P < 0.05 and ^**^P < 0.01. All experiments were repeated at least three independent experiments.

Bioinformatics website assays revealed that miR-214-3p interacted with circ_0001006 (Figure 5A). To identify the association of miR-214-3p with circ_0001006, we conducted RNA pull down assays using a circ_0001006-specific probe. circ_0001006 precipitation complex performed the enrichment of miR-214-3p (Figure 5B and 5C). Luciferase assay also confirmed that miR-214-3p could interact with circ_0001006 (Figure 5D). Then we found that the level of miRNA-214-3p was upregulated after transfecting sh-circ_0001006 and downregulation after overexpression circ_0001006 (Figure 5E and 5F). These results performed that circ_0001006 could bind to miR-214-3p.

PAK family is divided into two categories. I class PAK1-3, II class contains PAK4-6. Previous reports showed that mice knockout of PAK1 promote isoproterenol-induced cardiac hypertrophy, which could activate Erk1/2 and inhibit protein phosphatase 2A [19]. PAK family members participated in severe heart disease progression [20], and we observed that PAK6 possessed the potential binding site of miR-214-3p by using Target Scan Human 7.2 bioinformatics website (Figure 5G). The luciferase report verified that miR-214-3p could interact with and inhibit the expression of PAK6 (Figure 5G). Further, miR-214-3p reduced PAK6 expression, and circ_0001006 could induce the expression of PAK6 (Figure 5H–5K).

### Circ_0001006 regulates hypertrophy by targeting miR-214-3p/PAK6

**Figure 6:**
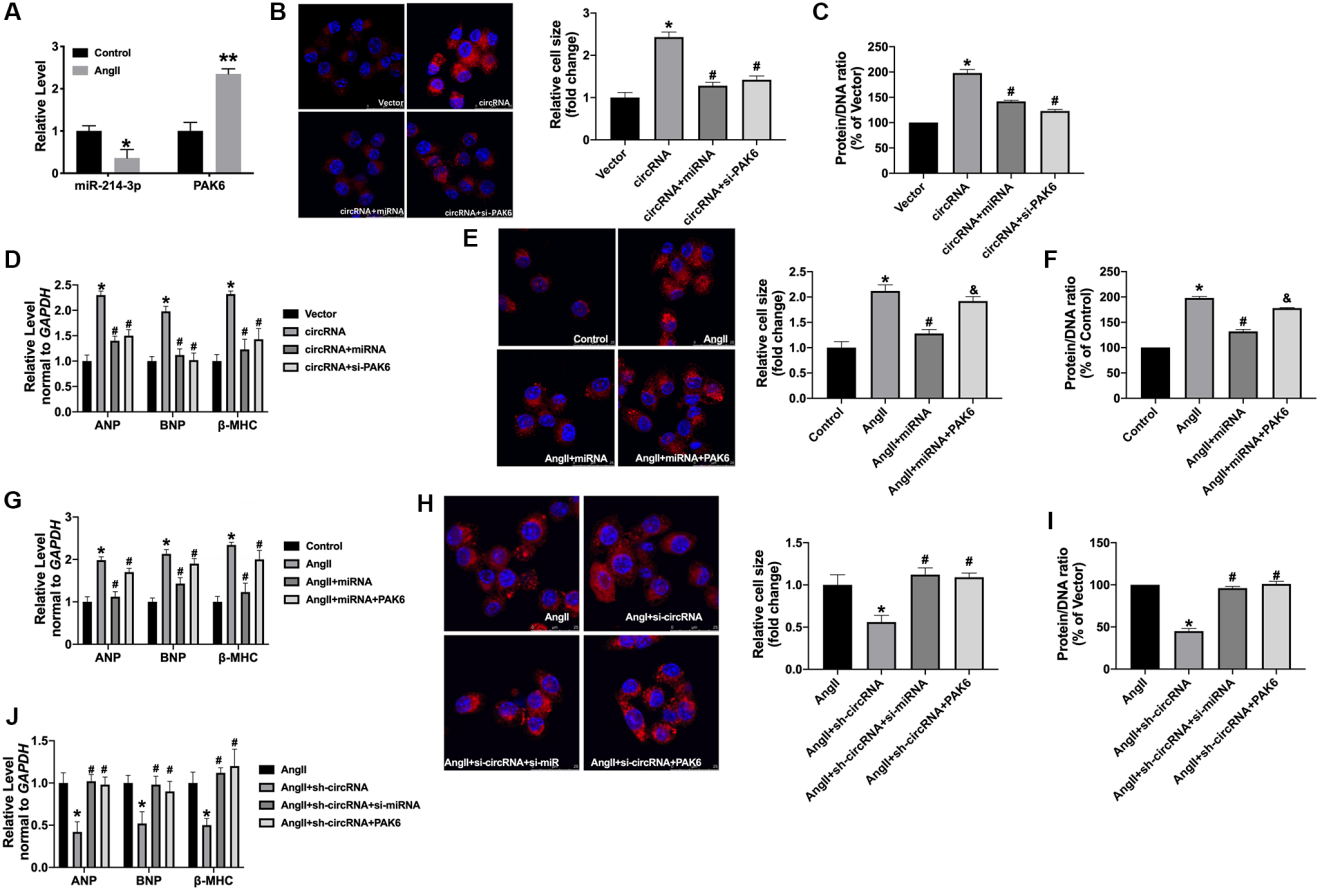
**The circ_0001006 regulates cardiac hypertrophy via suppressing miR-214-3p/PAK6.** (**A**) qRT-PCR assay analysis of PAK6 and miR-214-3p level in cardiomyocytes. (**B**) Representative immunofluorescence results of cardiomyocytes (The scale bar is 25 μm). (**C**) Protein/DNA ratio of the cardiomyocytes. (**D**) The expression of ANP, BNP, and β-MHC in cardiomyocytes. (**E**) Representative immunofluorescence results of cardiomyocytes (The scale bar is 25 μm). (**F**) Protein/DNA ratio of the cardiomyocytes. (**G**) The expression of ANP, BNP, and β-MHC in cardiomyocytes. (**H**) Representative immunofluorescence results of cardiomyocytes (The scale bar is 25 μm). (**I**) Protein/DNA ratio of the cardiomyocytes. (**J**) The expression of ANP, BNP, and β-MHC in cardiomyocytes. Data are shown as mean ± SEM. ^*^P and *^#^P* < 0.05 and ^**^P < 0.01. All experiments were repeated at least three independent experiments.

Next, we found the decreased level of miR-214-3p and increased level of PAK6 in AngII-treated cardiomyocytes (Figure 6A). Furthermore, we co-transfection circ_0001006 with miRNA-214-3p mimics or si-PAK6 in normal cardiomyocytes. The gain function of circ_0001006 increases the cell cross size and the protein/DNA ratio in Ang II-treated cardiomyocytes, which were remitted by miRNA-214-3p mimics or si-PAK6 (Figure 6B and 6C). The mRNA levels of hypertrophy biomarkers were also recovered by overexpression miRNA-214-3p or si-PAK6 (Figure 6D). Co-transfection of PAK6 abolished the function of miR-214-3p on Ang II-treated cardiomyocytes, which was performed by promoting hypertrophic markers expression (Figure 6E–6G). Meanwhile, silencing of circ_0001006 inhibited cardiomyocyte hypertrophy, which was abolished by si-miR-214-3p or PAK6 overexpression (Figure 6H–6J). Taken together, circ_0001006 regulates hypertrophy by targeting miR-214-3p/PAK6.

### Circ_0001006 prevents cardiac hypertrophy *in vivo*

**Figure 7:**
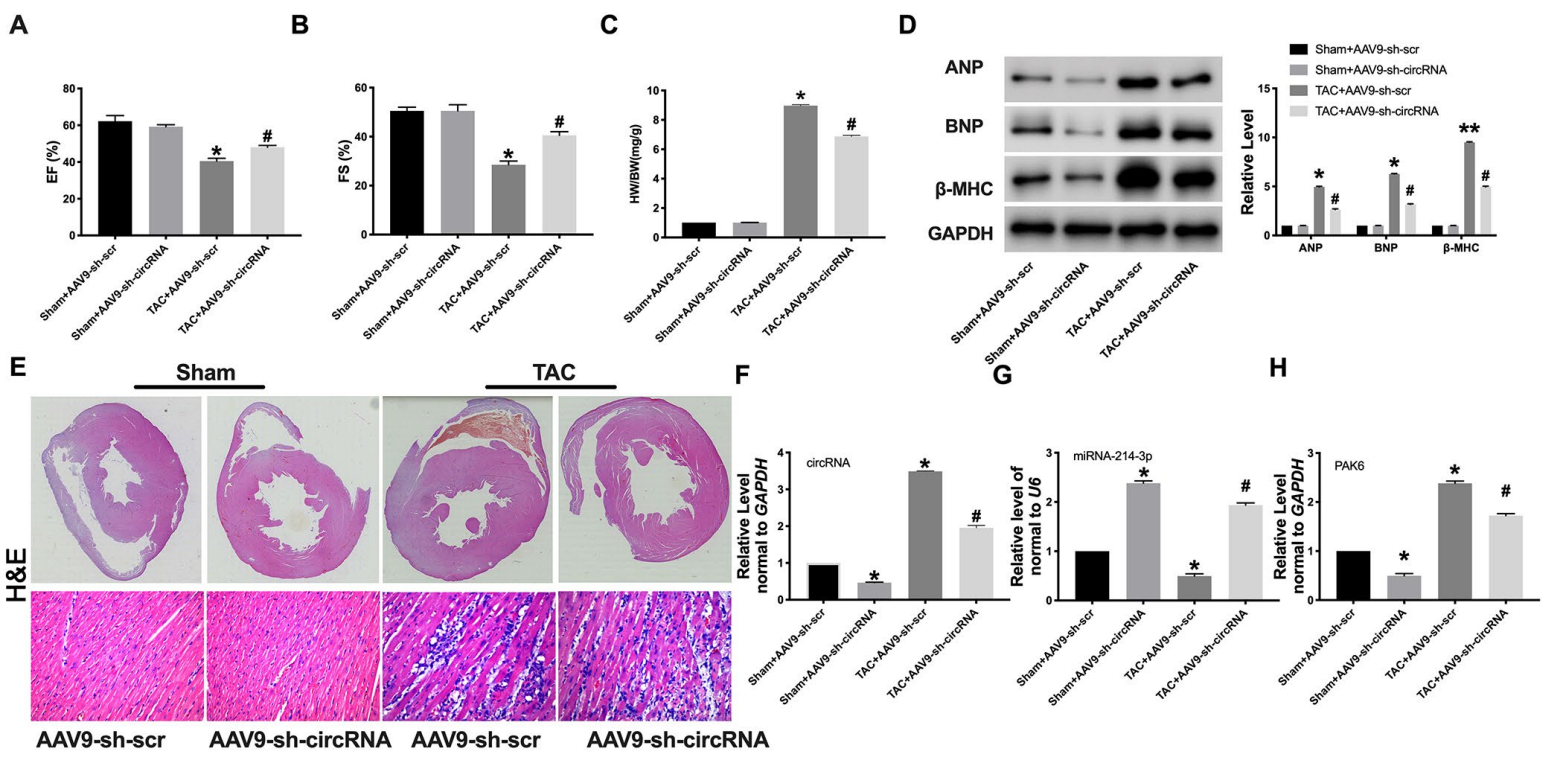
**Knockdown of circRNA_0001006 alleviated cardiac hypertrophy *in vivo.*** (**A** and **B**) EF (%) and FS (%) were assessed by echocardiography. (**C**) The histogram results of HW/BW ratios in different groups. (**D**) Western blot assay analysis of ANP, BNP, and β-MHC expression level in the tissue. (**E**) Representative histological results of the (**H** and **E**) Staining of the mouse heart tissues. (**F**) The AAV9 transfection efficiency of circ_0001006 in the mouse heart. (**G**) The miR-214-3p level in heart tissue. (**H**) The mRNA level of PAK6 in mice. ^*^P < 0.05 and ^**^P < 0.01 vs. Sham+AAV9-sh-scr group, ^#^*P* < 0.05 vs. Sham+AAV9-sh-circRNA group. Data are shown as mean ± SEM. All experiments were repeated at three independent experiments.

Then, AAV9 sh-circ_0001006 or AAV9 sh-Scr were injected into mice via tail vein for 21 days, and the mice have subjected to TAC surgery for creating cardiac hypertrophy model. The cardiac function was determined by echocardiography. AAV9 sh-circ_0001006 recovered the EF (%) and FS (%) in TAC mice (Figure 7A and 7B). The results showed a lower HW/BW ratio in the sh-circ_0001006 injection TAC mice (Figure 7C). The ANP, BNP, and β-MHC levels were dramatically downregulated when compared with the sh-Scr group in TAC mice (Figure 7D). H&E staining results performed that AAV9 sh-circ_0001006 prevented cardiac hypertrophy in TAC mice (Figure 7E). We performed qRT-PCR assays to verify the downregulation of circ_0001006 (Figure 7F). We also performed the change of miR-241-3p and PAK6 level was consistent with the results *in vitro* (Figure 7G and 7H). Together, these data suggest that circ_0001006 promoted cardiac hypertrophy via suppressing miR-214-3p targeting to PAK6.

## DISCUSSION

In conclusion, our research demonstrated that circ_0001006 could promote cardiac hypertrophy by decreasing miR-214-3p via targeting PAK6, which is a novel mechanism in cardiac hypertrophy. Our findings may provide a prediction and diagnosis method for pathological cardiac hypertrophy.

Pressure load-induced cardiac hypertrophy was mediated by different signaling pathways. Signaling pathways are numerous, link between each other, and form a complex network. Exploring the occurrence and development of cardiomyocyte hypertrophy is of profound significance for the prevention and treatment of myocardial hypertrophy.

In this research, we found that circ_0001006 was markedly upregulated in cardiac hypertrophy. We firstly investigated the role of cardiomyocytes with the gain or loss function of circ_0001006. We found that the gain function of circ_0001006 induced hypertrophy in cardiomyocytes, knockdown of circRNA performed opposite results. We demonstrated that circ_0001006 interacted with miR-214-3p to induce the expression of in cardiomyocytes. The present study revealed that circ_0001006 could induce cardiac hypertrophy via inhibiting miR-214-3p binding to PAK6.

Due to its unique molecular biological characteristics, circRNA plays a crucial role in the diagnosis of a variety of diseases. However, the fact that circRNAs are highly maladjusted in condition and exhibit a high degree of tissue and disease specificity makes them ideal candidates for disease diagnosis. CircRNA also plays a decisive function in heart disease. It was reported that circRNA_000203 suppressed miR-26b-5p/miR-140-3p to abolish the inhibition of Gata4, which aggravates cardiac hypertrophy [21]. As an endogenous sponge, circSlc8a1 can interact with miR-133a in cardiomyocytes, which indicated circSlc8a1 might play a key role in cardiac hypertrophy [22].

In previous reports, circ_0001006 was identified as a potential biomarker and therapeutic target in breast cancer brain metastasis [23]. In the plasma of gastric cancer patients, circ_0001006 may be used as diagnostic indicators due to anomalous change [24]. Up to now, studies of circRNA in heart disease, especially in cardiac hypertrophy, are limited, which requires us to explore the characteristics and mechanisms further. Our data report that circ_0001006 interacted with miR-214-3p. Functionally, circ_0001006 could promote the increases of cell cross area and the level of biomarkers, ANP, BNP, and β-MHC in Ang II-treated cardiomyocytes by targeting miR-214-3p.

Our current results have provided proof to sustain the viewpoint that miR-214-3p suppresses cardiac hypertrophy by repressing PAK6. TargetScan website analyses performed that PAK6 may possess the binding site of miR-214-3p, for further verify the supposes, luciferase report was conducted to perform miR-214-3p binding with 3′-UTRs of PAK6. The gain function of miR-214-3p repressed transcriptional level of PAK in cardiomyocytes. Then, PAK6 inhibited the anti-hypertrophy effect of miR-214-3p in cardiomyocytes, the conclusion showing that PAK6 participated in miR-214-3p-mediated cardiac hypertrophy progress.

## METHODS

### Animal models

All mice were fed under standard conditions, with standard food and water. Adult male C57BL/6 mice (~25 g, 8 weeks) previously described were used in this study [25]. Cardiac hypertrophy was induced by TAC (transverse aortic constriction) operation. The similar surgery without ligation was conducted in the sham group. Then the mice were fed for 4 weeks. Before 1 week of TAC surgery, the animals were injected with AAV9 sh-Scr or sh-circ_0001006 (Biocobio, Tianjin, China) was injected in the left ventricle myocardium. The effect of AAV9 reaches highest about 3 weeks. The animal study was reviewed and approved by Baotou Medical College.

### Microarray assay

TRIzol reagent (Invitrogen) was used to extract total RNAs from heart tissues, followed by the quantified analysis (NanoDrop). After the standard protocol of Arraystar, we conducted the microarray hybridization. Briefly, fluorescent complementary RNAs (cRNAs) were produced from the purified RNAs and hybridized in circRNA array. The arrays were scanned by Agilent Scanner G2505C and the images and the data were analyzed by gilent Feature Extraction software (version 11.0.1.1) and R software, respectively. The differently expressed circRNAs were shown in the heat map based on the criteria (fold change >2 and *P* < 0.05).

### Echocardiography

The cardiac function was analyzed by echocardiography measurement with a Vevo 2100 Imaging System furnished with a 30 MHz phased-array in mice with TAC for 8 weeks. Diastolic and systolic volumes were acquired by applying Simpson’s rule of discs to the serially acquired short-axis images (Table 1).

**Table 1:** Comparison of left ventricular echocardiographic parameters between the two groups.

**Index**	**8 weeks**	
	**Sham**	**TAC**
**LVIDs**	3.08 ± 0.21	4.09 ± 0.13
**LVIDd**	3.79 ± 0.23	4.92 ± 0.21
**LVPWd**	0.58 ± 0.03	0.92 ± 0.03
**EF**	54.23 ± 3.73	38.79 ± 3.73
**FS**	32.23 ± 4.23	23.79 ± 2.23

### Cell culture and treatment

Cardiomyocytes were collected from neonatal mice (1–3 days) [26]. Digesting the cardiac tissue with trypsin (Solarbio, Beijing, China), and cardiomyocytes were cultured at 37°C with 5% CO_2_ in DMEM (Waltham, USA) containing FBS (10%, Cromwell, USA) and penicillin/streptomycin (100 μL/mL, Sigma-Aldrich, USA). For inducing cell hypertrophy, the cardiomyocytes were disposed of with angiotensin II (Ang II, 200 nmol/L) for 48 hrs.

### Immunostaining

Cells were fixed with 14% formaldehyde and embedded in paraffin. Immunohistochemical staining was performed according to the instructions of the primary antibody (α-actinin, Abcam, Cambridge, UK). The nucleus was stained using DAPI. The images were observed by an optical microscope (Olympus, Japan).

### Protein/DNA ratio detection

The cells were washed using PBS and subjected in the cellular protein and DNA content quantitative analysis. The sample was centrifuged (10,000g, 10 minutes) after the treatment of 0.2 N perchloric acid (1 ml), followed by the incubation of NKOH (250ml, 0.3N, 60°C, 20 minutes). The Lowry method was used to analyze protein content, in which serum albumin served as the standard. The Hoechst dye 33258 was used to analyze DNA content, in which salmon sperm DNA served as the standard.

### Quantitative real-time PCR

Cells were transfected with vectors and/or oligonucleotides indicated in each experiment. RNA, and were treated with Trizol reagent (Sigma, USA) to isolate total RNA. The RNA was reverse-transcript to cDNA by using a superscript reverse transcriptase (Invitrogen, Carlsbad, CA), and quantified by a ViiA7 Quantitative PCR System (Applied Biosystems, Carlsbad, CA). The relative change of RNA levels was calculated by 2^–ΔΔCT^ method and normalized to GAPDH or U6.

### Dual-Luciferase reporter assay

The wild type sequences or mutant sequence of circ_0001006 and 3′UTR region of PAK6 were cloned into the pmirGLO vectors (Promega, USA). The cells were transfected with circ_0001006-WT and Mut, or PAK6-WT and Mut, along with miR-214-3p or NC. After 48 hours incubation, the cells were lysed and the luciferase intensity was detected by a Dual Luciferase assay kit (Biovision, China).

### Western blot analysis

Cells with the indicated treatment were lysed by ice-cold RIPA buffer (Thermo, USA) added with a cocktail of protease inhibitors (Thermo). The lysates were then subjected to the SDS-PAGE and shifted to nitrocellulose membranes (absin, China). The protein bands were interacted with primary antibodies at room temperature for 2 hours and 4°C overnight, followed by secondary anti-mouse and anti-rabbit antibodies. The bands were then visualized by using an Odyssey in an Odyssey software (LI-COR, USA).

### Histological analysis

The heart tissue was gathered and fixed in 4% paraformaldehyde for 24 hrs. Then the fixed tissues were embedded in paraffin. Next, a Paraffin slicer machine was used to cut slices (5-μm cross-sectional). H&E staining was used to evaluate cardiac morphology. The results were calculated with image J 6.0.

### Biotin-coupled circRNA and miRNA enrichment

Biotin-coupled miRNA and circRNA pull-down assays were executed according to the protocol [27, 28]. Briefly, HEK293 cells were transfected with 3′ end biotinylated circ_0001006 (20 nM) for 1 day. 4 MyOne streptavidin C1 Dynabeads was carried out to pull down biotin-coupled RNA complexes. The enrichment of circ_0001006 or miR-214-3p was measured by PCR.

### Statistical analysis

All data is statistical as a mean ± S.E.M. We performed Student’s *t*-test or a one-way ANOVA for statistical analysis. *P* < 0.05 was described as statistically significant.
